# Molecular Markers for Breast Cancer: Prediction on Tumor Behavior

**DOI:** 10.1155/2014/513158

**Published:** 2014-01-28

**Authors:** Bruna Karina Banin Hirata, Julie Massayo Maeda Oda, Roberta Losi Guembarovski, Carolina Batista Ariza, Carlos Eduardo Coral de Oliveira, Maria Angelica Ehara Watanabe

**Affiliations:** Laboratory of Polymorphism and Application Study of DNA, Department of Pathological Sciences, Biological Sciences Center, State University of Londrina, 86057-970 Londrina, Brazil

## Abstract

Breast cancer is one of the most common cancers with greater than 1,300,000 cases and 450,000 deaths each year worldwide. The development of breast cancer involves a progression through intermediate stages until the invasive carcinoma and finally into metastatic disease. Given the variability in clinical progression, the identification of markers that could predict the tumor behavior is particularly important in breast cancer. The determination of tumor markers is a useful tool for clinical management in cancer patients, assisting in diagnostic, staging, evaluation of therapeutic response, detection of recurrence and metastasis, and development of new treatment modalities. In this context, this review aims to discuss the main tumor markers in breast carcinogenesis. The most well-established breast molecular markers with prognostic and/or therapeutic value like hormone receptors, *HER-2* oncogene, Ki-67, and p53 proteins, and the genes for hereditary breast cancer will be presented. Furthermore, this review shows the new molecular targets in breast cancer: *CXCR4*, caveolin, miRNA, and *FOXP3*, as promising candidates for future development of effective and targeted therapies, also with lower toxicity.

## 1. Introduction

The global importance of cancer is unquestionable, considered the second cause of death worldwide. The incidence of different cancers had increased both in developed and in developing countries as a result of increasing exposure to risk factors and life expectancy [1]. Breast cancer is one of the most common cancers with more than 1,300,000 cases and 450,000 deaths each year worldwide [2]. In Brazil, 52,680 new cases of breast cancer are expected for 2012, with an estimated risk of 52 cases per 100,000 women [3].

Breast tumors are classified histologically according to the location of origin. The ductal tumors develop in breast ducts and represent 80% of tumors. The lobular tumors develop inside the lobes and account for 10 to 15% of cases. Other subtypes represent less than 10% of cases diagnosed per year [4]. Patients with invasive ductal carcinoma present higher lymphatic involvement and worse prognosis than less common types of breast carcinoma [5]. The staging system widely used is Tumor-Node-Metastasis (TNM) classification of malignant tumors, as recommended by the Union for International Cancer Control (UICC), which is an anatomically based system that records the primary and regional nodal extent of the tumor and the absence or presence of metastases. The evaluation of these parameters allows the determination of staging varying from stages 0 to IV [6].

The development of breast cancer involves a progression through series of intermediate processes, starting with ductal hyperproliferation, followed by subsequent evolution to carcinoma in situ, invasive carcinoma, and finally into metastatic disease [7]. Given the variability in clinical progression of disease, the identification of markers that could predict tumor behavior is particularly important in breast cancer. Also, the determination of tumor markers is a useful tool for the clinical management of cancer patients, assisting in diagnostic procedures, staging, evaluation of therapeutic response, detection of recurrence and distant metastasis and prognosis [8], helping in the development of new treatment modalities [9]. Therefore, this review aims to discuss the main tumor markers for breast cancer development, progression and possible new therapeutic targets.

## 2. Molecular Markers

According to US National Institutes of Health's (NIH) Working Group and Biomarkers Consortium, a molecular marker is a characteristic that is objectively measured as an indicator of pathogenic or normal biological processes, or a pharmacological response to a therapeutic intervention [10]. Although the most of these markers is protein, recently, gene expression patterns and altered DNA identified in tumor tissue have also taken prominence as tumor markers [11].

It is known that breast cancer represents a complex and heterogeneous disease that comprises distinct pathologies, histological features, and clinical outcome. Also, it is well established that this neoplasia has well-defined molecular subgroups based on gene expression profiling closely related to the behavior of these molecular subtypes. Sotiriou and Pusztai [12] pointed out that results from studies of gene-expression profiling have altered the view of breast cancer and provided a new tool for molecular diagnosis. Actually, the status of estrogen receptor (ER), progesterone receptor (PR), and human epidermal growth factor receptor type 2 (HER2) has been used as predictive markers for identifying a high-risk phenotype and for selection of the most efficient therapies [13].

The heterogeneity of breast cancer was reflected in array-CGH (comparative genomic hybridization) data of several reports, demonstrating clear or less clear associations with its subtypes [14]. After the sequencing of human genome and the technical progress in protein identification, it is reasonable considering an integrated program of genomics and proteomics to accomplish better comprehension of breast cancer features and the development of improved therapeutics [15]. Together, these results strengthened evidence of improved sensitivity and resolution methodologies, which contribute to the classification of breast cancer.

Within this context, in this review will be presented some well-stablished molecular markers of therapeutic value in the prognosis of breast cancer, and promising new markers not routinely used in clinical practice.

## 3. Well-Established Prognostic and/or Therapeutic Breast Cancer Markers

### 3.1. Hormone Receptors (HR)

Approximately more than one million women are diagnosed with breast cancer each year and approximately 700.000 of these have positive (+) hormone receptors (HR) [16]. The hormone receptors are expressed proteins both in the epithelium and in breast stroma which bind to circulating hormones, mediating their cellular effects [17, 18].

The HR best studied in breast cancer are estrogen receptor (ER) and progesterone receptors (PR). Breast cancers classified by positive immunohistochemistry (IHC) expression of ER and PR have different clinical, pathological, and molecular characteristics [19]. It is postulated that risk factors are closely associated with breast tumors ER+ and PR+ and may involve mechanisms related to exposure to estrogen and progesterone, while etiology of breast cancer ER− and PR− should be independent of hormone exposure [20, 21]. The ER and PR are highly associated with patient age at diagnosis, rising continuously with age.

During the 1980s, Tamoxifen became the first anti-estrogenic therapy targeted to ER for adjuvant therapy [22]. The antagonist effects of this drug in breast tissue may result from its ability to bind to the ligand-binding domain of ER, effectively blocking the potential for estrogen stimulation. Tamoxifen binding further prevents critical ER conformational changes that are required for the association of coactivators [23]. This therapy produced clinical remission in patients with breast cancer positive for ER, differently from tumors with low or undetectable levels of these receptors [24]. Additionally, tumor cells expressing hormone receptors presented a better response to hormone therapy and patients demonstrated higher survival, both disease free as overall [25, 26], and better prognosis [27]. Although hormone therapy has revolutionized the management of breast cancer and results have improved substantially in these patients, the optimal management remains a significant challenge.

### 3.2. Human Epidermal Growth Factor Receptor 2 (HER-2)

Several names has been given for this gene, such as c-erb-2, cerbB-2, C-erbB-2, HER-2, HER-2/neu, ERBB2, erbB2, erbB-2, neu/c-erbB-2/oncogene neu, neu protein, and neu [27]. This review will adopt the term “HER-2.” The HER-2 has been extensively studied in breast cancer since Slamon et al. [28] demonstrated an association between HER-2 amplification and poor prognosis [29].

HER-2 is a transmembrane tyrosine kinase receptor belonging to a family of epidermal growth factor receptors structurally related to epidermal growth factor receptor (EGFR), encoded by *ERBB2/HER2* oncogene located on chromosome 17q21 [30]. This oncogene is amplified in 20 to 30% of breast cancers and is considered a marker of poor prognosis, once its overexpression is associated with an aggressive phenotype of tumor cells, resistance to anti-hormonal, cytotoxic therapies, and low overall survival.

In the cell signaling, the homodimerization or heterodimerization of HER family receptors activates intracellular tyrosine kinase domain which promotes the autophosphorylation of tyrosine residues of cytoplasmatic tail and thus triggers pathways that results in survival and cellular proliferation [31]. However, according to crystallographic analysis, HER-2 is ready in binding conformation even in the absence of ligand, explaining why this receptor lacks natural ligands [32].

Currently, the humanized monoclonal antibody Trastuzumab, directed against the extracellular domains of HER-2, is indicated for the treatment of HER-2 positive breast cancer cases. The efficacy of Trastuzumab as part of an antitumor protocol has been validated in several clinical studies, where this antibody showed inhibitory effect on tumor growth and chemotherapy sensitizer [33]. Although the mechanisms by which Trastuzumab inhibits the signaling mediated by HER-2 are not fully understood, its antitumor effects are supposed to be conferred by inhibition of receptor-receptor interaction, receptor decreasing by endocytosis, blockade of extracellular domain cleavage of receptor, and activation of antibody-dependent cellular cytotoxicity (ADCC) [34, 35]. In addition to Trastuzumab, other therapeutic strategies have been developed to target HER-2 protein, such as tyrosine kinase inhibitor Lapatinib, which showed improved efficacy after failure of Trastuzumab therapy [36].

HER-2 status of breast cancer is routinely assessed by either IHC analysis of HER-2 protein or fluorescent in situ hybridization (FISH) analysis of gene copy number in primary tumor tissues. It was shown that HER-2 extracellular domain (ECD) can be shed into circulation by proteolytic cleavage from the full-length HER-2 receptor, and it is detected in serum of women with benign breast disease, primary and metastatic breast cancer [37]. The “soluble” receptor can be quantified by enzyme-linked immunoabsorbent assay (ELISA) method [38]. Tan et al. [39] established a Dot blot method to detect serum HER-2 levels, which is a valid and inexpensive assay with potential application in monitoring breast cancer progression in clinical situations.

Although HER-2 is associated with aggressive form of cancer, a specific subgroup named triple negative breast cancer (TNBC) arouses special interest, once they are orphan of directed treatment. TNBC is a subtype characterized by the lack of ER, PR, and HER-2 expression and it is associated with younger age at diagnosis [40]. There is an exhaustive search effort to find the drivers of this breast cancer subtype, because the usual antiendocrine and anti-HER2 targeted therapies are ineffective and traditional cytotoxic chemotherapy seems to be insufficient [41]. The aggressive clinical course, poor prognosis, and lack of specific therapeutic options have intensified current interest in this subtype of tumor [42]. The clinical behavior of TNBC is classically more aggressive than other types, like luminal A and B molecular subtypes, that according to Sørlie et al. [43] are considered of best and intermediate prognosis, respectively.

### 3.3. Ki 67 Antigen

The Ki-67 antigen, first described in 1983, is a labile, nonhistone nuclear protein that is tightly linked to the cell cycle and is expressed in mid-G1, S, G2, and M phases of proliferating cells but not in quiescent or resting cells of the G0 and early G1 phases. Ki-67 score is the most often measured on histological sections by IHC methodology and is defined as the percentage of stained invasive carcinoma cells [44, 45]. Vielh et al. [46] demonstrated a strong correlation between phase S and Ki-67 and they verified that quantitative evaluation of Ki-67 can offer a precise estimation of tumor proliferation index. According to the St. Gallen Consensus of 2011, the proliferation index is considered low or negative, when there are 14% or less stained nuclei and it is considered positive or high, when there are more than 14% of stained nuclei [47].

Biological markers that can predict a clinic or pathologic response to primary systemic therapy of early form, during a cycle of chemotherapy, can show considerable clinical importance. Patil et al. [48] evaluated Ki-67 index and apoptotic index (AI) before, during and after neoadjuvant chemotherapy with anthracycline in indigenous woman with breast cancer, but found no significant differences.

Tawfik et al. [49] demonstrated for the first time that high expression of Ki-67 in axillary lymph nodes but not in breast tissue is significantly associated with shorter patient survival. Based on this result, patients with higher proliferative activity in lymph nodes metastases might require more aggressive therapy and closer clinical monitoring of their disease.

The prognostic and predictive value of Ki-67 was evaluated in a review developed by Luporsi et al. [50], and they concluded that this biomarker could be considered as a prognostic factor for therapeutic decision; however, standardization of techniques and scoring methods are needed for integration of this biomarker in everyday practice.

### 3.4. Tumor Protein p53

The p53 is involved in several critical pathways including cell cycle arrest, apoptosis, DNA repair, and cellular senescence, which are essential for normal cellular homeostasis and genome integrity maintenance. Alteration of *TP53* gene or posttranslational modification in p53 protein can alter its response to cellular stress. The molecular archaeology of *TP53* mutation spectrum generates hypotheses concerning etiology and molecular pathogenesis of human cancer [51]. In breast cancer, approximately 30% of patients display *TP53* gene mutation, but this frequency fluctuates from more than 80% in basal-like to less than 15% in luminal-A subtypes [52].

According to Allred et al. [53], expression of mutant p53 protein was associated with high tumor proliferation rate, early disease recurrence, and early death in node-negative breast cancer. Dumay et al. [54] investigated *TP53* mutations in breast tumors from the luminal, basal, and molecular apocrine molecular subgroups. They found that subgroups differ not only in *TP53* mutation frequency but also in mutation types and consequences. They detected a high prevalence of missense mutations in luminal tumors and truncating mutations in basal tumors. In apocrine molecular tumors, despite high prevalence of insertions/deletions, p53 truncation was not increased. The observations point to different mutational mechanisms, functional consequences, and/or selective pressures in different breast cancers subtypes.

Mutations in *TP53* gene result in altered molecular conformation and prolonged protein half-life leading to nuclear accumulation of altered p53 protein. The IHC method detects this abnormal accumulation and acts as an indirect indicative of mutation in *TP53* gene [55]. This nuclear accumulation is an indicator of a poor clinical outcome for breast cancer patients. However, despite its prognostic value, there is still no proper treatment that takes into account the status of this marker.

### 3.5. Carbohydrate 15-3 and Carcinoembryonic Antigens (CA 15-3 and CEA)

Breast cancer is generally no longer curable once metastases are detected by “classical” means: clinical manifestations of the spread, imaging methods, and serum marker assays, such as those based on carcinoma antigen 15-3 (CA 15-3) or carcinoembryonic antigen (CEA) [56].

CA 15-3 in combination with CEA is also relevant tumor markers in breast cancer [57]. According to Geraghty et al. [58], the serum marker CA 15-3 has superior prognostic relevance in relation to CEA, but unlike these authors, Ebeling et al. [59] reported that prognostic value of CEA is higher than that of CA 15-3, which demonstrated that this marker has conflicting implications in breast carcinogenesis.

The CEA is a glycoprotein which has been shown to be expressed in vast majority of human colorectal, gastric, and pancreatic cancers, as well as in breast carcinomas and nonsmall cell lung carcinomas [60]. Determination of CEA in breast cancer is indicative of tumor size and nodal involvement. Therefore, CEA concentrations greater than 7.5 *μ*g/L are associated with high probability of subclinical metastases [61]. Prognosis of patients whose CEA level was within the normal range at the time of diagnosis is significantly better than those with elevated CEA levels [62].

CA 15-3 peptides are shed or soluble forms of MUC-1, which exists as a transmembrane protein consisting of two subunits that form a stable dimer. The release has been shown to be mediated by 2 proteases, ADAM17, and MT-MMP1 [63, 64]. This is heterogeneously expressed on the apical surface of different normal epithelial cell types, but it is aberrantly overexpressed in 90% of breast cancer [65].

Sandri et al. [66] found a prognostic role for CA 15-3 within subgroups of patients with luminal B and HER-2 positive disease. According to their results, baseline CA 15-3 might be value in the identification of higher risk of relapse, where adjuvant chemotherapy must be introduced. In other words, this study showed explicitly that presence of an abnormal CA 15-3 presurgical value is associated with an increased risk of recurrence and death. Further studies using database analyses or prospective trials are required to confirm the prognostic value of presurgery CA 15-3 determination in breast cancer. If confirmed, the presence of elevated CA 15-3 should be added to the list of features that must be taken into account while making a proper treatment choice. According to Mendes et al. [67], measurement of tumor markers is a tool for detection of distant metastases, and the marker CA 15-3 seems more efficient when compared to CEA. Monitoring of breast cancer patients after surgical treatment using only this tumor marker is insufficient. However, simultaneous use of both serum markers (CA 15-3 and CEA) allows the early diagnosis of metastasis in up to 60–80% of patients with breast cancer [68].

### 3.6. Breast Cancer Susceptibility Genes (*BRCA1* and *BRCA2*)

Approximately 80% of the cases related to familial breast cancer are associated with one gene of hereditary susceptibility for breast and ovarian cancer, *BRCA1 *and *BRCA2. *The *BRCA* genes have been classified as tumour-suppressor genes, because the loss of wild-type allele has been observed in tumors of heterozygous carriers. BRCA proteins play important roles in different cellular processes, including activation and transcriptional regulation, repair of DNA damage, beyond the control of cell cycle, cellular proliferation, and differentiation [69, 70].

Besides breast cancer, these genes are associated with elevated risk of ovary, prostate, and pancreas cancers. However, despite its association with inherited predisposition, somatic disease-causing mutations in *BRCA1* or *BRCA2* are extremely rare in sporadic breast cancer [71, 72].

The frequency and spectrum of mutations within *BRCA1 *or *BRCA2* genes show considerable variation between ethnic group and geographic region, probably due to interactions between different lifestyle and genetic characteristics. Studies have discussed the role of maternal or paternal inheritance of *BRCA* mutation affecting risk of breast cancer. Shapira et al. [73] showed that lifetime risk was higher in *BRCA* mutation inherited from the father, compared to the mother. However, in accordance to Senst et al. [74], although the risk of breast cancer seems to be modestly higher in women with paternal *BRCA1* mutation, the results of the study were not significant. Thus, data are not sufficiently compelling to justify different screening recommendations for the two subgroups. Furthermore, parental mutation origin also did not affect the risk in women with *BRCA2* mutation.

Family history profiles can predict *BRCA1 *or *BRCA2 *mutation, mainly those characterized by first-degree relatives with ovarian cancer or breast cancer along with young age at diagnosis, bilateral occurrence and increased number of affected relatives. These predictors would be useful in genetic counseling and decision-making for a genetic test but they are still of limited value since a considerable number of *BRCA1 *or *BRCA2 *mutations are observed in breast cancer families without such risk factors. Definitive predictors need to be developed in future studies [75].

Genetic counseling and genetic testing to identify *BRCA1 *and *BRCA2 *gene mutations in high-risk patients are widely available and commonly employed in the US and Europe [76, 77]. Individuals who undergo genetic testing by sequencing their DNA for specific regions of these genes and discover that they carry a *BRCA *mutation can have the diagnosis of breast cancer anticipated and perhaps in some cases prevented. If a high-risk status of these women had been recognized, they might have had the opportunity to choose genetic counseling, testing, more effective cancer surveillance, and potentially preventive options, such as prophylactic surgery and/or chemoprevention [78]. Among these preventive options, bilateral mastectomy, although invasive, reduces approximately 90% the risk of breast cancer in women with *BRCA1*/*2* mutations [79].

According to Apostolou and Fostira [80], more susceptible genes have been discovered and BRCA1 and BRCA2 predisposition seems to be only a part of the story. These new findings include rare germline mutations in other high penetrant genes; the most important between them include TP53 mutations in Li-Fraumeni syndrome, STK11 (serine/threonine kinase 11) mutations in Peutz-Jeghers syndrome, and PTEN (phosphatase and tensin homolog on chromosome ten) mutations in Cowden syndrome. Furthermore, more frequent, but less penetrant, mutations have been identified in families with breast cancer clustering, in moderate or low penetrant genes, such as CHEK2 (checkpoint kinase 2), ATM (ataxia telangiectasia mutated), PALB2 (partner and localizer of BRCA2), and BRIP1 (BRCA1-interacting protein C-terminal helicase 1).

## 4. Future Candidate Markers for Prognosis and Therapeutic Responses in Breast Cancer Evolution

### 4.1. Proliferating Cell Nuclear Antigen (PCNA)

Moldovan et al. [81] have described the proliferating cell nuclear antigen (PCNA) as a nonhistone nuclear protein that forms a homotrimeric ring encircling DNA double helix and acts as a molecular platform to recruit proteins involved in DNA synthesis, such as DNA polymerase delta, cell-cycle control, DNA-damage response, and repair. PCNA exists in two distinct forms: replication-competent chromatin-bound form and chromatin unbound form, which is not involved in DNA synthesis [82]. For instance, it is not clear how these two populations of PCNA are regulated. In a number of tumors, measurement of this protein was associated with mitotic activity and tumor grade [83]. The PCNA signal transduction has an important impact on growth regulation of breast cancer cells and is associated with poor overall survival [84]. Recently, it was reported that phosphorylation of PCNA at tyrosine 211 (Y211) is a promising treatment target in prostate cancer [85]. The results obtained by Zhao et al. [84] suggested that targeting phospho-Y211 PCNA could be an effective strategy in breast cancer treatment as well in the future.

### 4.2. Caveolin

Studies showed that caveolae and caveolin 1 play an essential role in many molecular, cellular, and physiological processes [86]. Caveolae are special invaginated microdomains of the plasma membrane found in the majority of mammalian cells and serve as membrane organizing centers. Three members of caveolin family (CAV1, CAV2, and CAV3) have been identified and they play a pivotal role in intracellular trafficking of cellular components and in signal transduction [87].

Recently, it has been reported an strong association between CAV1 and CAV2 expression and high histological grade, and lack of hormone receptors positivity (ER and PR) in basal-like breast cancer subtype [88], providing evidence that these proteins can have oncogenic properties. Aside from breast, association between caveolin expression and poor patient outcome was noticed in other tumor tissues, including prostate [89], lung [90], and central nervous system [91].

The caveolin 1 influences cancer formation, progression, and prognosis, but this influence is not so sharp, in spite of recent results that have clarified many roles. Its role as oncoprotein or tumor suppressor may depend on interaction with molecular signaling molecules by specific regions, and this may be modified by genetic changes, mRNA, and protein expression level. Current and future research into mechanisms by which caveolin 1 function in tumorigenesis process will most likely lead to a new molecular marker in diagnosis and prognosis and even in treatment of breast cancer [86].

### 4.3. Receptor C-X-C Chemokine Receptor Type 4 (CXCR4)

The CXCR4 is transmembrane G-coupled receptor protein, identified as a coreceptor for T-cell line tropic strains of human immunodeficiency virus [92]. Its role in breast cancer metastasis was first documented in 2001 [93].

This receptor is required for migration of breast cancer cells from the primary site to lung, bones, and lymph nodes, which represent organs that secrete high levels of chemokine CXCL12. For this reason, CXCR4 has been found to be a prognostic marker in breast cancer, among other types of cancer [94].

Schioppa et al. [95] made an important observation that the expression of CXCR4 is up-regulated in tumor cells resulting from a change in tumor microenvironment. Tumor cells cultured in hypoxic conditions, for example, showed significant overexpression of CXCR4.

The expression level of CXCR4 is significantly correlated with lymph node metastasis [96]. Patients who had CXCR4 overexpression had significantly higher incidence of cancer recurrence and cancer-related deaths than those in low CXCR4 expression group [97]. Its expression also is significantly related to tumor size, advanced TNM stage, and shorter overall- and disease-free survival. However, in luminal or HER2-positive breast cancer groups, CXCR4 was not correlated with such clinic-pathological characteristics and survival. This association is cardinal in TNBC patients who expressed high levels of CXCR4, which have poorer disease-free survival and overall survival, compared with TNBC patients expressing low levels of this marker [98]. These findings indicated that CXCR4 high expression in TNBC might indicate a more aggressive tumor phenotype.

Therefore, this receptor may be a potential therapeutic target in cancer therapies for breast cancer. So far, the best studied among the compounds that inhibit CXCR4-CXCL12 interaction is the antagonist AMD3100. This compound significantly inhibits the invasion and metastasis activity of cancer cells [99].

### 4.4. Chemokine (C-C Motif) Ligands 2 and 5 (CCL2 and CCL5)

Many studies have addressed the involvement and roles of inflammatory chemokines CCL2 (MCP-1, membrane cofactor protein-1) and CCL5 (RANTES, regulated on activation, normal T-cell expressed and secreted) in breast malignancy. Belonging to chemokine super family, CCL2 and CCL5 are well recognized because of their activities in the immune context, where they induce leukocyte directed motility. Acting mainly in inflammatory reactions, these two chemokines stimulate migration primarily of monocytes and T-cells to damaged or infected sites [100–102].

Some studies have shown that CCL2 and CCL5 expressions are higher in breast cancer tissue than in corresponding normal tissue [103, 104]. CCL2 has been correlated with higher tumor grade and has been shown to have significant prognostic value for relapse-free survival. The CCL2 likely exerts its protumorigenic effects through recruitment of tumor-associated macrophages and angiogenesis [105]. Furthermore, CCL2/CCR2 (CCL2 receptor) chemokine signaling seems to be implicated in cell migration and its overexpression is associated with breast cancer metastasis to both lung and bone [104, 106].

The CCL5/CCR5 (CCL5 receptor) axis is active in patients affected by aggressive basal subtype of breast cancer. Murooka et al. [107] showed that CCL5 enhanced MCF-7 (breast cancer cell lines) proliferation. Furthermore, according to Velasco-Velazquez and Pestell [108], CCR5 promoted breast cancer invasiveness and metastatic potential. These results indicated that CCL5 expression is directly correlated with more advanced stage of disease, emphasizing their involvement in breast cancer progression [109]. In this context, these two chemokines could be considered as prognostic markers and therapeutic targets for breast cancer.

### 4.5. Growth Factors: EGF, HGF, IGF, VEGF, and TGF-*β*

The role of growth factors has been extensively analyzed both in cancer risk and tumor progression. Cerna et al. [110] found a negative correlation between insulin-like growth factor I (IGF1) and severity of cancer. Thus, according to these authors, this growth factor cannot be used for quick and correct orientation in clinical condition of patients in the early stages of tumor growth, unlike epidermal growth factor (EGF). Nevertheless, the IGF1 and EGF are stimuli to migration of cancer cells to distant areas, to form metastasis, and have been implicated in the development and progression of human breast carcinoma [111].

The hepatocyte growth factor (HGF) is considered a progression and aggressiveness marker of breast cancer and data obtained by Kucera et al. [112] fully corresponds to this. Based on their data, this marker could potentially be used as an additional tool for the differentiation between benign and malignant tumor. Ahmed et al. [113] also demonstrated that serum levels of HGF may help in the diagnosis of breast cancer patients and may aid in disease prognosis. However, Cerna et al. [110] related the opposite for HGF as well as IGF1 and vascular endothelial growth factor (VEGF). It was demonstrated that tumor stromal VEGF-A expression is a valuable prognostic indicator of breast cancer-specific and disease-free survival at diagnosis and can therefore be used to stratify patients with inflammatory breast cancer (IBC) into low-risk and high-risk groups for death and relapses. Furthermore, high levels of tumor stromal VEGF-A may be useful to identify IBC patients who will benefit from antiangiogenic treatment, since VEGF-A is the most potent promoter of angiogenesis and lymphangiogenesis [114].

Conflicting results have been published about the transforming growth factor-*β* (TGF-*β*). Oda et al. [115] showed that homozygous patients for CC genotype from T869C polymorphism presented a higher TGF-*β* expression and suggested a role of this gene as progression marker for breast carcinoma. It is known that overexpression of TGF-*β* by both tumor and stromal tissue can facilitate the development of metastasis, mainly in behalf of TGF-*β* stimuli to angiogenesis and increased tumor cell motility [116]. According to Sheen-Chen et al. [117], high TGF-*β*1 serum levels have been associated with advanced stages of breast cancer; thus, it may reflect the severity of invasive breast cancer. However, Figueroa et al. [118] found that TGF-*β* expression was correlated with favorable prognostic features. These results are seemingly discordant and possibly represent the dual role of TGF-*β* in cancer development, in which it displays both tumorigenic and tumor-suppressive effects. de Kruijf et al. [119] claimed that combining TGF-*β* biomarkers provides prognostic information for patients with stage I–III breast cancer. The authors believed that this marker could identify patients at increased risk for disease recurrence that might therefore be candidates for additional treatment.

### 4.6. V-Myc Myelocytomatosis Viral Oncogene Homolog (Avian) (*MYC*)

The *MYC* proto-oncogene family (comprising c-myc, N-myc, and L-muc) ranks among the most exhaustively studied group of genes in biology. MYC is a basic helix-loop-helix zipper (bHLHZ) protein whose activity is tightly regulated by its direct binding to another bHLHZ protein MAX. *MYC* activation can lead to transcriptional activation or repression of specific genes [120]. This gene seems to have an important role in carcinogenesis and tumor replication, growth, metabolism, differentiation, and apoptosis [121].

Amplification of *MYC *has been reported in breast cancer as well as in many other cancers. However, amplification of this gene is not established as prognostic or predictive factor yet because there are many inconsistent results [122, 123]. Notwithstanding, several converging studies have suggested that *MYC* may play an important function in breast cancer.

A recent report from Horiuchi et al. [124] found that TNBC tumors exhibit elevated *MYC* expression, resulting in increased activity of the *MYC* pathway. They showed that CDK inhibition effectively induced tumor regression, indicating that aggressive breast tumors with elevated *MYC* expression are uniquely sensitive to CDK inhibitors.

*MYC* amplification shows strong correlation with ER status, stage of disease (initial), and existence of distant metastasis and tends to be associated with high histologic grade, positive axillary nodal status, and a high S-phase fraction. Furthermore, its amplification is not significantly associated with overall survival of patients with invasive cancer. Thus, this genetic alteration is a feature of aggressive breast cancer, but is unlikely to be a clinically useful prognostic factor [122, 125].

Thus, despite that the expression of *MYC *is significantly different between breast cancer patients and healthy controls [126], correlation between *MYC* amplification and different clinicopathological parameters are inconsistent. Regardless of lack of evidence for the prognostic significance of *MYC* amplification, it could represent a clinically useful predictive parameter in metastatic breast cancer [122].

### 4.7. Forkhead Box Protein 3 (FOXP3)

Forkhead box protein P3 (Foxp3) plays a critical role in differentiation, development, maintenance, and function of regulatory T-cells (Treg) [127]. However, it does not necessarily confer a Treg phenotype when expressed in CD4+ T lymphocytes. High Treg levels have been reported in peripheral blood, lymph nodes, and tumor specimens from patients with different types of cancer. The precise mechanisms by which Tregs suppress immune cell functions remain unclear, and there are reports of both direct inhibition through cell-cell contact and indirect inhibition through the secretion of anti-inflammatory mediators such as interleukin. Signals from the microenvironment have a profound influence on the maintenance and progression of cancers. Although T-cells present the most important immunological response in tumor growth in the early stages of cancer, they become suppressive CD4+ and CD8+ regulatory T-cells after chronic stimulation and interactions with tumor cells, thus promoting rather than inhibiting cancer development and progression [128].

It was recently demonstrated that FOXP3 is expressed at both mRNA and protein levels in the nucleus of epithelial cells in prostate [129], breast [130, 131], and lung [132]. It is already becoming clear that cancer cells can show dysregulated FOXP3 expression. Several studies have examined the distribution of FOXP3 in normal and malignant epithelial breast cells [131, 133]. Each study has consistently reported that FOXP3 is expressed constitutively within the nucleus of healthy epithelial cells. However, description of FOXP3 localization in cancerous epithelia is less definitive. Some reports showed a nuclear location similar to that observed in healthy cells whereas others describe either a complete absence of FOXP3, mutations or a change in subcellular distribution [129, 131, 133]. When breast cancer survival rate was correlated with location of this marker, patients with FOXP3 restricted to cytoplasm had similarly poor prognosis than patients with no detectable FOXP3 [131]. These data suggested that failure recruitment of FOXP3 to the nucleus could act as an important prognostic marker associated with a more aggressive form of breast cancer and poor survival. Similarly, almost 20% of breast cancer cells and 80% of non-malignant cells expressed nuclear FOXP3, when assessing subcellular distribution of FOXP3 in breast cancer patient samples [133].

FOXP3 is able to repress the expression of MYC [129] and in normal breast epithelium is able to bind to and repress the expression of HER-2 [133], with prognostic relevance [33].

Overbeck-Zubrzycka et al. [134] demonstrated that normal breast epithelia expressed FOXP3 constitutively within nucleus and failed to express CXCR4, whereas breast cancer samples and breast cancer metastasis expressed diminished levels of nuclear FOXP3 and also expressed significantly higher levels of membrane CXCR4. The increased expression of CXCR4 on these cells allows a potent response to CXCL12 and migration to site-specific regions of the body [135, 136].

### 4.8. microRNA

Over the past decades, an increasing amount of evidence has demonstrated applications of microRNAs (miRNAs) as tissue based markers for classification and prognosis of several human cancers, including breast cancer [137, 138].

miRNAs are naturally occurring, noncoding small RNA molecules of 21–24 nucleotides that binds partially or completely to 3′untranslated regions (3′-UTRs) of protein-coding genes, leading to cleavage or translational repression of targets [139, 140]. The miRNA can be stably present in whole blood, serum, and plasma [141], but the origin of circulating miRNA is still unclear. It has been proposed that tumor-associated miRNAs can be released into bloodstream when tumor cells are dying and being lysed [142] or through active secretion of miRNA loaded exosomes by tumor cells [143].

Some studies have indicated that microRNAs play a pivotal role in most critical biological events, including development, proliferation, differentiation, cell fate determination, apoptosis, signal transduction, organ development, hematopoietic lineage differentiation, host-viral interactions, and tumorigenesis [144, 145].

Wu et al. [146] detected more than 800 miRNAs in the circulation of breast cancer patients. Two of them, miR-375 and miR-122, exhibited strong correlation with clinical outcomes, including neoadjuvant chemotherapy response and metastatic relapse. These results may allow optimized chemotherapy treatments and preventive antimetastasis intervention in future clinical applications. Wang et al. [147] demonstrated that miR-122 acts as a tumor suppressor and plays an important role in inhibiting tumorigenesis through targeting IGF1R and regulating PI3K/Akt/mTOR/p70S6K pathway.

In breast cancer specimens, miR-497 expression pattern was negatively correlated with pathological stage, lymphatic metastasis, tumor size, and HER-2, and no correlation was found between miR-497 and ER, PR and p53 status. The overexpression of miR-497 results in downregulation of Bcl-w (antiapoptotic member of the Bcl-2 family), causing cellular growth inhibition and apoptotic enhancement, as well as G0/G1 phase arrest, acting like a tumor suppressor. Thus, breast cancer patients with elevated expression of miR-497 have better prognosis, and this marker may turn out to be a new prognostic marker [148].

Genome-wide analyses have identified deregulated miRNA expression in human malignancies [149] and a potential dual role in tumor formation, highlighting that miRNAs can modulate oncogenic or tumor suppressor pathways, including *p53, c-MYC, RAS, *and* BCR-ABL*, while expression of miRNAs themselves can be regulated by oncogenes or tumor suppressors.

However, miRNA can be prejudicial to breast cancer patient, contributing to tumor development. For instance, miR-373 and miR-520c stimulate cancer cell migration and invasion in vitro and in vivo [150]. Many studies have demonstrated the potential of miRNAs, as regulators, and they may serve as novel diagnostic and prognostic candidates and potential therapeutic targets.

## 5. Conclusion

Breast cancer is the second leading cause of women mortality and morbidity worldwide and this cancer represents one of the most privileged malignancy regarding the use of markers with predictive values, but especially two therapeutic strategies of great clinical relevance are known and applied in patient routine, Tamoxifen and Trastuzumab. Otherwise, many women are still diagnosed in advanced stages of disease with poor evolution. Hence, an intense search for markers that may be crucial in the course of disease; especially those with prognostic and therapeutic purposes will be needed to develop personalized treatment. In this context, some of the molecules discussed in this review provided strong evidence that evaluation and application of these breast cancer markers will play a significant role in more effective and targeted therapies, with lower toxicity to patients.
